# Cold induction of nuclear FRIGIDA condensation in *Arabidopsis*

**DOI:** 10.1038/s41586-023-06189-z

**Published:** 2023-07-12

**Authors:** Zhicheng Zhang, Xiao Luo, Yupeng Yang, Yuehui He

**Affiliations:** 1 State Key Laboratory of Protein and Plant Gene Research, Peking-Tsinghua Center for Life Sciences, School of Advanced Agricultural Sciences, Peking University, Beijing, China; 2 Shanghai Center for Plant Stress Biology, Chinese Academy of Sciences Center for Excellence in Molecular Plant Sciences, Shanghai, China; 3 University of Chinese Academy of Sciences, Beijing, China; 4 Peking University Institute of Advanced Agricultural Sciences, Shandong Laboratory of Advanced Agricultural Sciences in Weifang, Weifang, China

**Keywords:** Transcription, Plant development

arising from: P. Zhu etal. *Nature* 10.1038/s41586-021-04062-5 (2021)

In over-wintering annuals of *Arabidopsis thaliana* grown in temperate regions, prolonged cold exposure in winter, through the physiological process of vernalization, represses the expression of the potent floral repressor *FLOWERING LOCUS C* (*FLC*) to enable the transition to flowering in spring^1,2^. Recently, Zhu et al.^3^ reported that cold induced nuclear condensation of FRIGIDA (FRI) for *FLC* repression and that cold-induced antisense RNA *COOLAIR* promoted FRI condensation during prolonged cold exposure. Here we report that the cold-induced formation of nuclear FRI condensates is independent of *COOLAIR*.

Before exposure to cold, *FRI* activates *FLC* expression, and long-term continuous winter cold exposure (typically lasting more than a month) results in *FLC* repression in *FRI*-bearing winter annuals grown at high latitudes^1,4^. Prolonged cold exposure induces the expression of *COOLAIR*, a group of non-coding antisense RNAs initiating from a region downstream of the 3′ end of *FLC* that is composed of proximally polyadenylated class I and distally polyadenylated class II transcripts^5^. *COOLAIR* expression reaches a high level after around 3 weeks of cold exposure and subsequently declines under constant cold temperature^6^. Using a CRISPR–Cas9 system, we previously constructed several lines in which a large part of the core *COOLAIR* promoter region was removed^7^, resulting in the elimination of both class I and class II *COOLAIR* transcripts before cold exposure (Fig. 1a,b and Extended Data Fig. 1a,b). Furthermore, consistent with a recent study^8^, cold induction of *COOLAIR* expression was eliminated in these core promoter deletion lines (Fig. 1b), partly because the *cis*-acting cold-responsive elements located in the promoter region have been removed. To examine the role of *COOLAIR* in FRI condensation, we introduced a functional *FRI-GFP* into two lines in which the *COOLAIR* promoter was deleted—*ΔCOOLAIR-1* and *ΔCOOLAIR-2*—in the rapid-cycling accession Col-0 (bearing a loss-of-function *fri* allele^9^) by genetic transformation. Subsequently, independent *FRI-GFPΔCOOLAIR* lines (numbers 2, 5 and 7) were backcrossed to Col-0 and *ΔCOOLAIR-1* or *ΔCOOLAIR-2*, respectively, resulting in F_1_ progeny of *FRI-GFPΔCOOLAIR*^*−/−*^ and *FRI-GFPΔCOOLAIR*^*+/−*^. In these lines, FRI–GFP is fully functional and acts to activate *FLC* expression before cold exposure (Extended Data Fig. 1c–f).

**Fig. 1:**
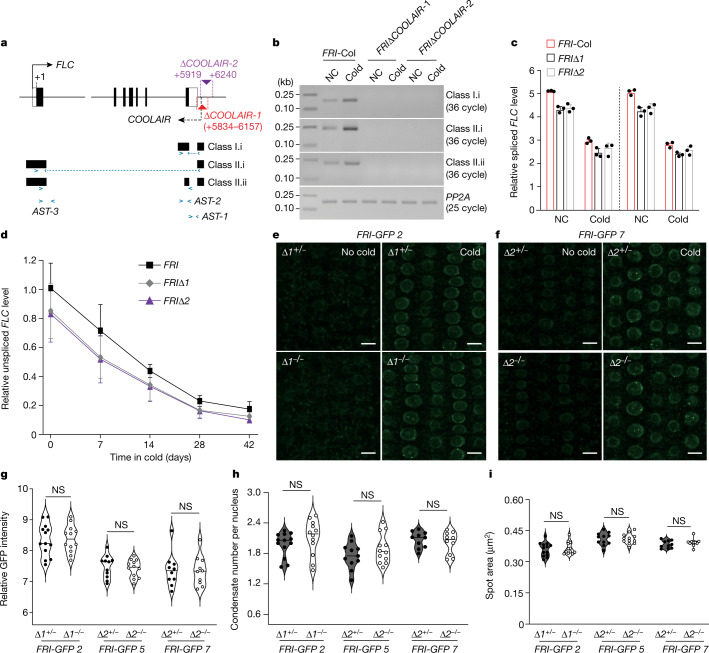
Functional analysis of *COOLAIR* in cold-mediated nuclear FRI condensation and *FLC* repression. **a**, Schematic of the *FLC* locus. The A of ATG is indicated (+1), the blue arrows show the primer positions and the dashed lines show the interexon primers. **b**, *COOLAIR* expression is eliminated in *FRIΔCOOLAIR* lines (*FRIΔCOOLAIR-1* (*FRIΔ1*) and *FRIΔCOOLAIR-2* (*FRIΔ2*)). Seedlings were exposed to cold for 14 days or no cold (NC). *FRI*-Col is a winter-annual reference line. The constitutively expressed *PP2A* (*At1G13320*; *PROTEIN PHOSPHATASE 2A SUBUNIT A3*) was used as an internal control. **c**,**d**, Quantification of spliced (**c**) and unspliced *FLC* (**d**) transcripts in cold-treated seedlings. Seedlings were cold-treated for 14 days and two biological replicates were conducted in **c**. The levels of *FLC* transcripts were normalized to *PP2A*. Data are mean ± s.d. of three technical replicates (**c**) or biological replicates (**d**). The relative expression to *FRI*-Col (before cold exposure) is presented in **d**. **e**,**f**, Confocal microscopy images of FRI–GFP in the root tip nuclei of *FRI-GFP* 2 (**e**) and *FRI-GFP* 7 (**f**) seedlings. Seedlings were treated with cold for 14 days. Scale bars, 10 μm. **g**, The fluorescence intensity of FRI–GFP in the root tips treated with cold for 14 days. Data points are plotted on bar graphs. **h**,**i**, Quantification of FRI–GFP condensates (condensate number per nucleus (**h**) and spot area (**i**)) in the nuclei of cold-treated root tips. For **g**–**i**, 10–13 seedlings per F_1_ population were scored and data were analysed using two-tailed *t*-tests with Welch’s correction; NS, not significant (*P* > 0.05). Source data

We next determined whether the loss of *COOLAIR* expression might reduce nuclear FRI condensation. We measured the fluorescence intensity of FRI–GFP in the root tips and the size and number of FRI–GFP condensates in root tip nuclei of cold-treated *FRI-GFPΔCOOLAIR*^*+/−*^ and *FRI-GFPΔCOOLAIR*^*−/−*^ seedlings and found that there was no difference between these two genotypes (Fig. 1e–i). Furthermore, we crossed a *FRI-GFP* line^3^ to both *ΔCOOLAIR-1* and *ΔCOOLAIR-2* and obtained homozygous *FRI-GFPΔCOOLAIR* lines. Subsequently, we measured the size and number of FRI–GFP condensates in root tip nuclei in these lines after cold exposure and found that there was no statistically significant difference (Fig. 2a–f). Together, these results show that the cold-induced formation of nuclear FRI condensates is independent of *COOLAIR* expression, given that, before and during cold exposure, *COOLAIR* expression (including class I and class II transcripts) was eliminated in both the *ΔCOOLAIR-1* and *ΔCOOLAIR-2* lines.

**Fig. 2:**
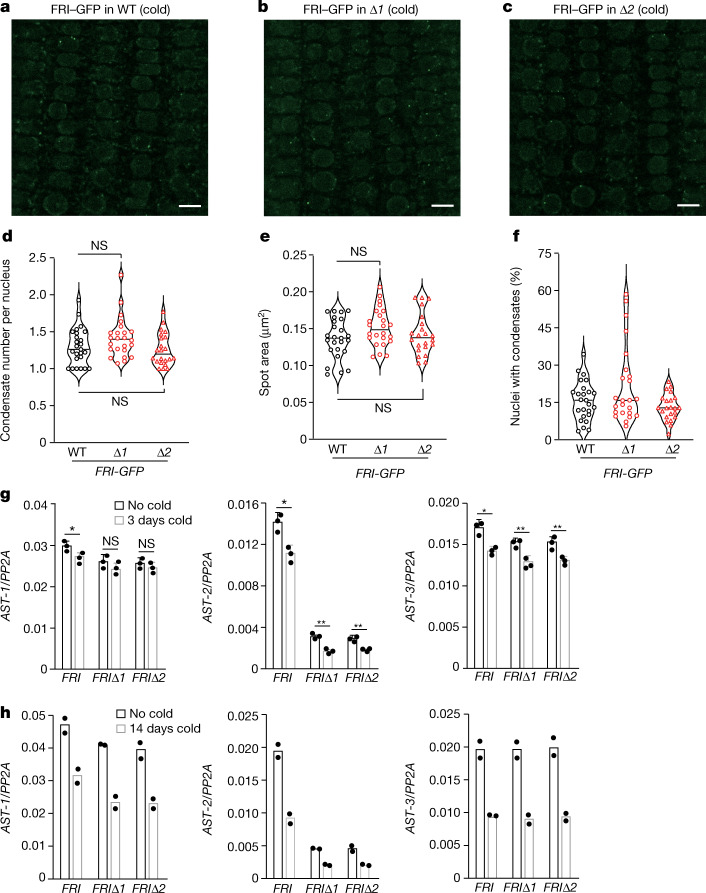
Characterization of nuclear FRI–GFP condensation and antisense transcription at *FLC* in the absence of *COOLAIR* expression. **a**–**c**, Confocal microscopy images of FRI–GFP in root-tip nuclei from the indicated seedlings (WT (**a**), Δ*COOLAIR-1* (**b**) and Δ*COOLAIR-2* (**c**)) exposed to cold for 14 days. Scale bars, 10 μm. **d**–**f**, Quantification of FRI–GFP condensates (number (**d**), spot area (**e**) and percentage of nuclei with condensates (**f**)) in the nuclei of cold-treated root tips. 22–25 seedlings per line were scored. For **d** and **e**, statistical analysis was performed using two-tailed *t*-tests with Welch’s correction. **g**,**h**, Quantification of ASTs at *FLC* in the indicated seedlings exposed to cold for 3 days (**g**) and 14 days (**h**). The examined regions with ASTs are indicated in Fig. 1a. AST-3 is known as CAS (convergent antisense transcript). For **g**, data are mean ± s.d. of three biological replicates. For **g**, statistical analysis was performed using two-tailed *t*-tests; *P < 0.05, **P < 0.01. Data points in **h** denote two biological replicates. Source data

Zhu et al.^3^ reported that, in the cold, the FRI protein enriched class II.ii *COOLAIR* transcripts and that the FRI–class II.ii interaction was closely connected with cold-induced FRI condensation. Using a transgenic *FLC* terminator exchange (*TEX*) line in which the *COOLAIR* promoter was replaced with an *RBCS3B* (encoding Rubisco small subunit 3B) terminator, Zhu et al.^3^ found that the size and number per nucleus of FRI–GFP condensates were reduced in the *TEX* line compared with those in the non-transgenic background (Col-0). In the *ΔCOOLAIR* lines that we used in this study, the class II transcripts are eliminated before cold exposure and in the cold. Thus, we conclude that the FRI-class II.ii interaction and *COOLAIR* expression are not involved in cold-induced FRI condensation. The cause for the discrepancy between the two studies is unclear.

In addition to the *COOLAIR* transcripts initiated downstream of the 3′ end of *FLC*, there are other antisense transcripts (ASTs) initiated within the *FLC* locus (see, for example, ref. ^10^). We examined these ASTs in *FRI*-Col (a reference winter-annual line^11^) and *FRIΔCOOLAIR* seedlings, and found that they were at low levels before cold exposure (Fig. 2g). After cold exposure for 3 days, the expression of ASTs in all three examined regions declined in *FRI*-Col, and was apparently reduced in two examined regions in the *FRIΔCOOLAIR* seedlings (Fig. 2g); cold exposure for 14 days strongly suppressed the expression of ASTs in both the *FRI*-Col and *FRIΔCOOLAIR* lines (Fig. 2h). Thus, in contrast to *COOLAIR*, the expression of ASTs is repressed along the early phase of long-term cold exposure or vernalization. The function of ASTs in the vernalization-mediated *FLC* repression remains to be seen.

Cold induction of *COOLAIR* expression in the early phase of vernalization was reported to mediate *FLC* repression^5,6^. We measured the levels of *FLC* transcripts (both spliced and unspliced) in the cold-treated *FRIΔCOOLAIR* seedlings, and found that loss of *COOLAIR* expression in either *FRIΔCOOLAIR-1* or *FRIΔCOOLAIR-2* had no effect on the progression of transcriptional shutdown of *FLC* during cold exposure or on post-cold stable silencing of *FLC* (Fig. 1c,d and Extended Data Fig. 1g), consistent with observations in a recent study^8^. Notably, in our vernalization study, like several other studies reporting a role of *COOLAIR* for *FLC* repression^5,6^, seedlings were exposed to a constant cold temperature, the mechanisms uncovered through which may not fully represent *FLC* regulation by winter cold in the fields with fluctuating cold temperatures.

In summary, our study shows that the cold-induced formation of nuclear FRI condensates is independent of *COOLAIR*. Moreover, our vernalization study with constant cold temperature shows that *COOLAIR* is not involved in *FLC* repression by prolonged cold exposure. Thus, more in-depth experiments would be required to resolve the role of *COOLAIR* in vernalization.

## Methods

*Arabidopsis thaliana FRI*-Col, Col-0, *ΔCOOLAIR-1* and *FRIΔCOOLAIR-1* were described previously^7^. Treatment of the seedlings with constant cold and quantification of the expression of genes of interest using quantitative PCR were performed as previously described^12^. *COOLAIR* expression was examined by semiquantitative PCR, after reverse transcription using transcript-specific primers (5′-TGGTTGTTATTTGGTGGTGTGAA-3′ for class I; and 5′-GCCCGACGAAGAAAAAGTAG-3′ for class II^10^). A list of the PCR primers is provided in Extended Data Table 1.

*FRI*_*pro*_*:FRI-GFP* was constructed by cloning a 4.8 kb genomic *FRI* fragment (2.5 kb promoter plus the 2.3 kb entire coding region without the stop codon) upstream of the *GFP-*coding region in the binary vector pMDC110^13^. Microscopy analysis and image quantification of nuclear FRI–GFP condensates were performed as follows. Root tips of the seedling samples were imaged using the Zeiss LSM900 confocal microscope with a ×40/1 NA water objective and an Airyscan detector of GaAsP-PMT. GFP fluorescence was excited at a wavelength of 488 nm (argon ion laser and laser power 8.0%), and detected at 490–620 nm in lambda mode. All of the images were obtained with a pixel size of 0.119 μm, and exported using the ZEN3.1 software (Zeiss) for quantitative analysis. The number of spots (with an area of larger than 0.1 μm^2^) per nucleus and the spot area in the F_1_ seedlings were obtained by outlining the spots using Graphics from ZEN3.1. Similarly, the fluorescent spots with an area of larger than 0.05 μm^2^ were scored in the root tips of the seedlings bearing the homozygous *FRI-GFP*^3^.

## Reporting summary

Further information on research design is available in the Nature Portfolio Reporting Summary linked to this Article.

## Online content

Any methods, additional references, Nature Portfolio reporting summaries, source data, extended data, supplementary information, acknowledgements, peer review information; details of author contributions and competing interests; and statements of data and code availability are available at 10.1038/s41586-023-06189-z.

### Supplementary information

Reporting Summary

### Source data

Source Data Fig. 1

Source Data Fig. 2

**Extended Data Fig. 1:**
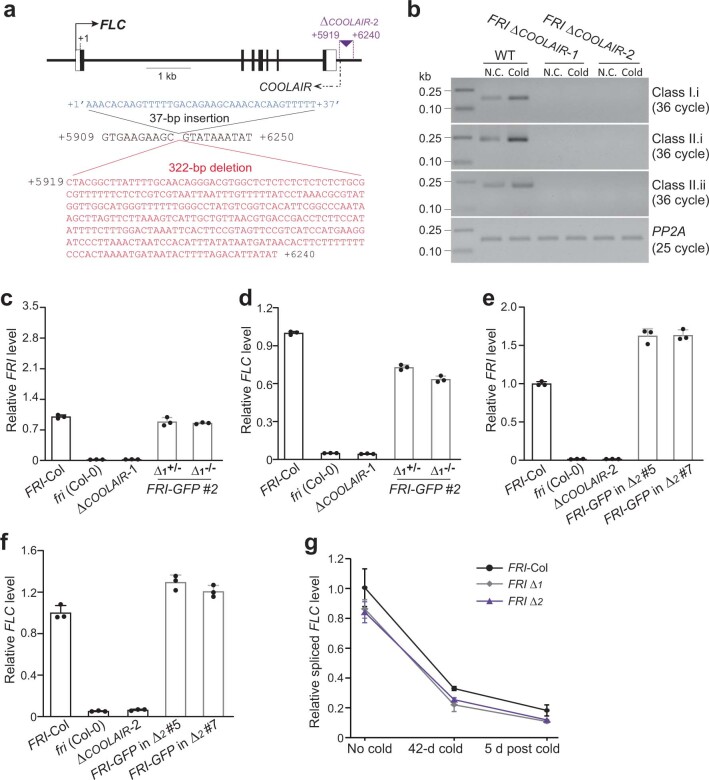
Characterization of *COOLAIR* promoter deletion lines and functional analysis of *FRI-GFP*. **a**, Genomic sequences around the 322-bp promoter deletion in the *ΔCOOLAIR*-2 line. Notably, there is a 37-bp insertion at +5919. **b**, *COOLAIR* expression is eliminated in the *COOLAIR* promoter deletion lines. Shown is a biologically-independent repeat experiment of Fig. 1b. **c**–**f**, Relative *FRI* (**c**,**e**) and *FLC* (**d**,**f**) expression in the indicated seedlings. Transcript levels of *FRI* and *FLC* (spliced) were normalized to *PP2A*. Relative expression to *FRI*-Col is presented. Individual data points are shown. Error bars for s.d. of three biological replicates. **g**, Relative levels of spliced *FLC* transcripts upon 42-d cold exposure. 5-d post cold, return to warmth for 5 d after 42-d constant cold exposure. Error bars for s.d. of three biological replicates. Source data

Source Data Extended Data Fig. 1

## Figures and Tables

**Extended Data Table 1:**
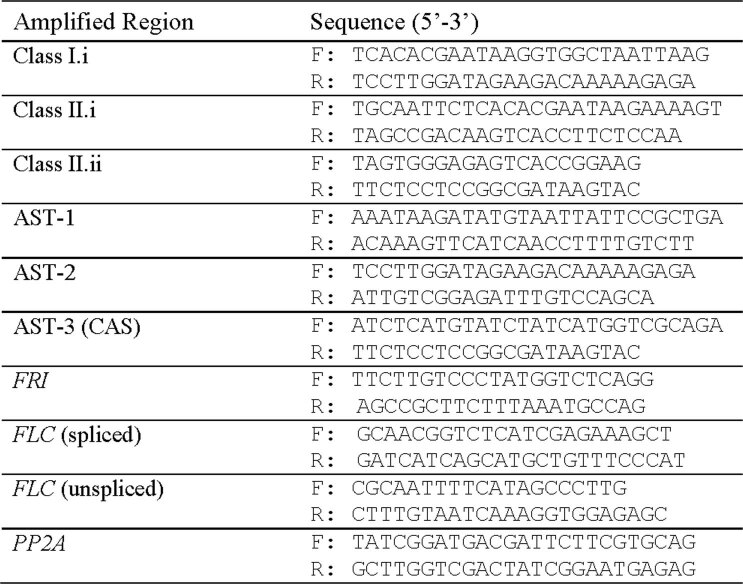
Primers used in the study

## Data Availability

Source data are provided with this paper.
